# Advanced Diabetes Management Using Artificial Intelligence and Continuous Glucose Monitoring Sensors

**DOI:** 10.3390/s20143870

**Published:** 2020-07-10

**Authors:** Martina Vettoretti, Giacomo Cappon, Andrea Facchinetti, Giovanni Sparacino

**Affiliations:** Department of Information Engineering, University of Padova, 35131 Padova, Italy; martina.vettoretti@dei.unipd.it (M.V.); cappongi@dei.unipd.it (G.C.); facchine@dei.unipd.it (A.F.)

**Keywords:** continuous glucose monitoring sensor, artificial intelligence, decision support system, prediction, optimization, personalized therapy, diabetes

## Abstract

Wearable continuous glucose monitoring (CGM) sensors are revolutionizing the treatment of type 1 diabetes (T1D). These sensors provide in real-time, every 1–5 min, the current blood glucose concentration and its rate-of-change, two key pieces of information for improving the determination of exogenous insulin administration and the prediction of forthcoming adverse events, such as hypo-/hyper-glycemia. The current research in diabetes technology is putting considerable effort into developing decision support systems for patient use, which automatically analyze the patient’s data collected by CGM sensors and other portable devices, as well as providing personalized recommendations about therapy adjustments to patients. Due to the large amount of data collected by patients with T1D and their variety, artificial intelligence (AI) techniques are increasingly being adopted in these decision support systems. In this paper, we review the state-of-the-art methodologies using AI and CGM sensors for decision support in advanced T1D management, including techniques for personalized insulin bolus calculation, adaptive tuning of bolus calculator parameters and glucose prediction.

## 1. Standard Type 1 Diabetes Therapy and Margins for Improvement Using New Technologies

Type 1 diabetes (T1D) is a chronic metabolic condition caused by the autoimmune destruction of pancreas beta-cells which are responsible for insulin production. Since insulin is the hormone stimulating blood glucose (BG) absorption by the body tissues, people with T1D, if not properly treated, present persistently high BG concentrations. 

T1D requires constant management through frequent BG self-monitoring, diet, physical exercise and exogenous insulin administration: both the timing and the dose of insulin must be accurately tuned. Insulin underdosing increases the risk of hyperglycemia, i.e., BG concentration >180 mg/dL, which if prolonged may lead to the incidence of diabetes-related complications, such as nephropathy, retinopathy, cardiovascular diseases and neuropathy [1,2,3]. On the other hand, insulin overdosing can drive in short time to hypoglycemia, i.e., BG concentration <70 mg/dL, which if prolonged and severe can increase the risk of seizure, coma or even death.

In standard T1D insulin therapy, a basal insulin dose is administered to maintain BG concentration in a safe range when the patient is fasting (e.g., during the night or during the day if a meal is skipped). In addition, a bolus dose, called a meal bolus, is taken before each meal to face the subsequent rise in BG concentration commonly produced by food intake. If abnormally high BG levels are present after the meal, the patient may take an additional bolus, called a correction bolus, to bring back the BG concentration in the safe range. Patients are instructed to calculate the dose of insulin boluses by themselves, either manually or with the help of a software/app called a bolus calculator (BC) [4,5], which usually adopts a simple equation that includes some patient-specific parameters to personalize a bolus computation. These parameters, as well as the basal insulin dose, are established by an expert (e.g., diabetologist or certified diabetes educator), to achieve the best fit for a patient’s insulin requirements. Usually, an initial value for the therapy parameters is established by resorting to empirical rules based on general patient characteristics, for example age and body weight [6]. Then, the value of these therapy parameters will be manually adjusted during routine visits, based on a retrospective analysis of each patient’s glucose concentration measurements. Patients are often advised to use different therapy parameter values at different times of the day, since the insulin sensitivity of an individual may vary during the day according to a circadian pattern [7]. Furthermore, insulin sensitivity may be influenced by daily activities (e.g., physical activity), illness, stress, hormonal cycles and other factors, thus insulin therapy parameters continually need to be revised. 

The above clearly reveals the burden the daily management of T1D imposes. Patients need to monitor their BG concentration several times a day and carry out consequent therapeutic actions, such as determining insulin doses, or when hypoglycemia is detected, ingesting a small amount of fast-acting carbohydrates (15–20 g), the so-called hypotreatment. In standard T1D therapy, glucose monitoring is performed through portable devices for self-monitoring of BG (SMBG) that measure BG concentration using a small drop of capillary blood collected by finger-prick. Notably, the American Diabetes Association recommends monitoring BG before meals and snacks, at bedtime, occasionally after meals too, prior to exercise, when the patient suspects hypoglycemia, after treating hypoglycemia until glucose concentration returns in normoglycemia, and also prior to critical tasks, such as driving [8]. As a result of the dozens of decisions and actions required every single day, the total number of routine operations for T1D therapy management can be between 100,000 and 500,000 in a patient’s lifetime. Mitigating this exceptional burden, at least in part, today seems possible through the advent of new, minimally invasive, devices for glucose monitoring and insulin delivery.

Specifically, in the last 20 years, continuous glucose monitoring (CGM) sensors were developed. CGM sensors measure interstitial glucose concentrations in subcutaneous tissue almost continuously (e.g., every 1–5 min) for several consecutive days or weeks [9]. CGM sensors revolutionized, de facto, interstitial glucose monitoring and opened up new, exciting scenarios in the daily management of diabetes [10]. Indeed, in contrast to SMBG, CGM sensors allow to track glucose dynamics and they ensure prompt detection of even asymptomatic high/low glucose events, which are difficult to capture with SMBG.

CGM sensors can be divided into two main categories: Professional CGM sensors and real-time CGM sensors [11,12]. Professional CGM sensors are prescribed by healthcare professionals usually for limited periods of time, they record glucose concentration data in blinded modalities (i.e., the patient cannot visualize the data in real-time) and they allow the healthcare professional to retrospectively review the patient’s glycemic trends and make therapy adjustments. Conversely, with real-time CGM sensors (rtCGM) the recorded data are accessible in real-time to the patient, who can use these data for improved decision-making in the daily management of T1D. 

The most popular rtCGM sensors are minimally-invasive electrochemical sensors that measure interstitial glucose concentration by a small transcutaneous electrode placed under the skin of the abdomen, or the arm. The glucose oxidation in proximity of the sensor generates a current signal, which is then converted to a glucose concentration profile [13]. The sensor transmits glucose measurements to a portable receiver (this can be a standalone device or a smartphone app) that the patient can use to inspect the sensor’s readings in real-time. Every 7 to 14 days, depending on the sensor type, the needle electrode must be substituted. Besides minimally-invasive sensors, implantable rtCGM sensors are now marketed both in Europe and U.S. [14]. These systems consist of an implantable fluorescence sensor, which is inserted in the subcutaneous tissue of the upper arm by a medical team through a small surgical operation. Then a removable transmitter, attached to the skin with an adhesive, sitting directly over the sensor, stores the glucose data, alerts when glucose values are out of the target range and transfers the data to a Smartphone app.

Another category of glucose sensors is the one of flash glucose monitoring (FGM) systems [15], which are similar to rtCGM sensors with the difference that they can show glucose data only when the receiver is scanned over the sensor.

The use of rtCGM sensors has proved to be very beneficial for glycemic control [10,16,17]. Indeed, rtCGM devices are able to provide, in real-time, not only the glucose concentration present but also information about the glucose trend (typically displayed to the user by an arrow), which can be used for making more informed therapeutic decisions. Moreover, rtCGM devices provide vibratory or auditory alerts when hypo/hyperglycemic events are detected, i.e., when the glucose concentration measured exceeds pre-set, customizable, thresholds for hypoglycemia and hyperglycemia [10]. Real-time high and low glucose alerts have been recently introduced also in FGM systems [18]. High and low glucose alerts allow the patient to react promptly to an abnormal glucose concentration level, e.g., in the case of low glucose by taking a hypotreatment or, in the case of high glucose, a correction insulin bolus. Since 2015, some rtCGM devices have also received regulatory approval for therapeutic use, which means that rtCGM measurements may be used for making therapeutic decisions, such as insulin dosing [19].

In addition to glucose monitoring systems, patients with T1D can also benefit from the use of insulin delivery devices. Insulin pumps are wearable medical devices that inject rapid-acting insulin into the subcutaneous tissue (for example, of the abdomen, the back, or the arm) almost continuously through a cannula connected to a refillable reservoir of insulin in the pump. Remarkably, some insulin pumps can be integrated with rtCGM sensors into the so-called closed-loop (or artificial pancreas) system, in which a control algorithm automatically adjusts the insulin dose based on the glucose concentration measured [20,21]. Nevertheless, the use of an insulin pump may prove to be burdensome for some patients [22], and the majority of patients with insulin-dependent diabetes still rely on multiple daily injection (MDI) therapy, in which the patient manually injects insulin doses using insulin pens or syringes. A new generation of insulin pens for MDI therapy was introduced in 2017, smart insulin pens, which are able to record the amount and timing of each insulin dose and wirelessly transmit the information, via Bluetooth, to a dedicated mobile app [23]. Smart pens can also provide the user with reminders should a dose be missed, with potential benefits for glycemic control.

The increasing availability of information offered by wearable devices, such as CGM sensors, insulin pumps, smart pens, and activity trackers, is making the decision-making process increasingly complex. Current research in diabetes is putting considerable effort into developing algorithms and software applications to enhance T1D therapy by exploiting the latest technologies and their interoperability. One of these applications is decision support systems (DSSs) for advanced diabetes management [24], i.e., tools that can assist the patient and/or the doctor during the decision-making process by automatically analyzing the patient’s data and providing personalized recommendations about therapy adjustments. The large amount of data that can be acquired from patients with diabetes makes artificial intelligence (AI) techniques particularly attractive for developing diabetes DSSs [25].

The aim of this paper is to provide a narrative overview of the latest progresses in the development of DSSs for advanced T1D management using AI and rtCGM sensor data. Although in this review, we focus on rtCGM sensors, which we will henceforth call simply CGM, most of the concepts apply to FGM as well. In Section 2, the concept of DSS for advanced T1D management will be presented. Section 3, Section 4 and Section 5 will review the state-of-the-art algorithms proposed for the 3 main tasks of a DSS for advanced T1D management, that are personalized insulin bolus calculation, adaptive tuning of BC parameters and glucose prediction. Note that this article is not a systematic literature review, rather a narrative review of those state-of-the-art AI-based approaches that, according to our experience as investigators active in diabetes technology research, are most relevant in the field of T1D DSSs for patient use.

## 2. Decision Support Systems for Advanced Diabetes Management

Early DSSs for diabetes management were designed to support health care providers in the retrospective analysis of a patient’s data and therapy revision either during face-to-face visits or, remotely, in a telemedicine framework [26,27]. More recently, the availability of wearable devices that display the data to the patient in real-time, such as CGM sensors, has permitted the development of DSSs targeted to the patient. In other words, DSSs that can provide recommendations regarding therapeutic decisions directly to the patient. These DSSs for patients offer an alternative to closed-loop systems. Indeed, many users do not feel confident about using the artificial pancreas [28,29], as they are concerned about possible errors occurring in the insulin pump and so prefer an open-loop therapy (i.e., a therapy in which the patient manually sets the insulin doses), which can be assisted by a DSS. Moreover, while the artificial pancreas requires the patient to wear an insulin pump, the DSS is suitable for both pump and MDI users. 

**Figure 1:**
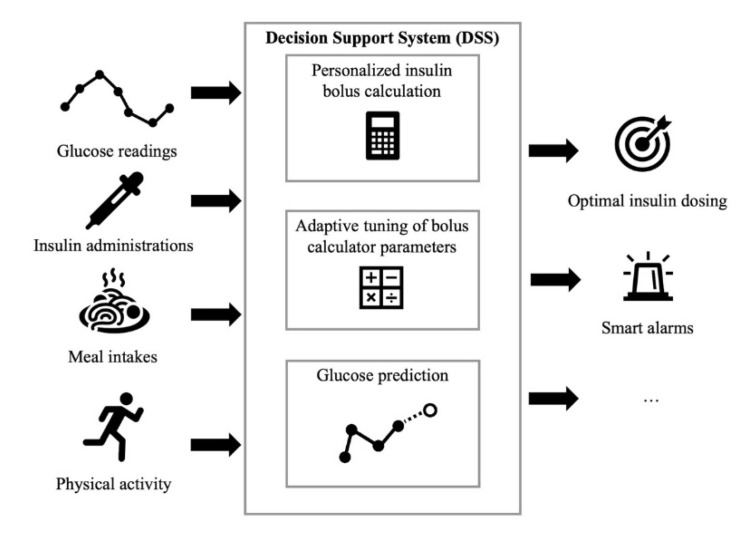
A general structure of a DSS for patient use. By using information on glucose concentration, insulin administrations, meal intakes and physical activity, a DSS provides recommendations on optimal insulin dosage and several other useful features to help patient in managing diabetes. A DSS usually implements three modules: Personalized insulin bolus calculation (Section 3), adaptive tuning of bolus calculator parameters (Section 4) and glucose prediction (Section 5).

The DSSs for patient use proposed in the literature receive input information about glucose concentration, diet, insulin administrations and physical activity and primarily perform three tasks: Personalized insulin bolus calculation, adaptive tuning of BC parameters and glucose prediction (Figure 1). For example, Breton et al. [30] proposed a DSS that includes: A CGM-based bolus advisor, which optimally modulates insulin boluses based on an estimate of the patient’s current insulin sensitivity; an exercise advisor, which provides ad-hoc behavioral advice if the risk of hypoglycemia is detected when the patient begins an exercise session; and a retrospective insulin titration tool, based on a personalized simulation model, which periodically revises the therapy parameters if a glycemic risk profile is detected. The European Commission too is supporting research on the development of diabetes DSSs. For example, PEPPER (Patient Empowerment through Predictive PERsonalised decision support), a project funded by the European Commission under the Horizon 2020 program, recently developed a DSS for personalized T1D management that includes an adaptive bolus advisor, based on case-based reasoning, as well as a system for carbohydrate recommendations based on a predictive model [31]. Several diabetes technology companies are also working on commercial products for decision-support in diabetes management. DreaMed (DreaMed Diabetes Ltd., Petah Tikva, Israel) recently introduced Advisor Pro, a AI-based DSS for diabetes management that analyzes the patient’s data and provides personalized recommendations on the adjustment of therapy parameters [32]. However, this system is not designed to send recommendations directly to patients without them first having been approved by a clinician.

In the following sections, we will discuss in more detail the three main tasks of DSSs for patient use, laying particular emphasis on those methodologies which are most related to AI. The three tasks are: personalized insulin bolus calculation, adaptive tuning of BC parameters and glucose prediction. 

## 3. Personalized Insulin Bolus Calculation Based on Continuous Glucose Monitoring Sensors

In standard T1D therapy, the dose of insulin boluses (B) is calculated by patients themselves adopting the following simple equation:

(1) $$B=\frac{CHO}{CR}+\frac{G_{c}-G_{T}}{CF}-IOB.$$

In this formula, the first addendum is used for meal boluses only, and it represents the insulin amount required to cover the amount of carbohydrates in the meal. This is calculated as the ratio between the patient’s own estimate of carbohydrates in the meal (CHO) and a patient-specific parameter, called a carbohydrate-to-insulin ratio (CR), which represents how many grams of CHO are covered by each unit of insulin. The second addendum of Equation (1) corrects the insulin dose to account for BG concentrations above or below the target. This is calculated as the difference between the BG concentration measured by the patient at bolus time (G_c_) and the target BG concentration (G_T_), divided by the correction factor (CF), which is a patient-specific parameter that represents how much BG is lowered by each unit of insulin. Finally, the third addendum of Equation (1) subtracts the insulin on-board (IOB), i.e., the insulin amount of previously injected boluses that is still acting in the body, from the insulin dose. In the following, we will refer to Equation (1) as the standard formula (SF) for bolus computation. 

The SF was designed when SMBG was the only way to measure glucose concentration at home. In fact, the SF only uses information about the current glucose concentration (G_c_), ignoring the information on glucose rate-of-change (ROC) that is now available with modern CGM sensors. Researchers are currently exploring the possibility of improving the insulin dose calculation using the information on glucose ROC, which can easily be obtained from CGM. Indeed, if ROC is positive/negative (rising/falling glucose) it is intuitive to use this information to predict a future glucose concentration level higher/lower than the current one, and then to increase/decrease the amount of insulin computed through the SF. Following this rationale, several empirical methodologies that adjust the SF according to ROC have been proposed [33,34,35]. However, an in silico head-to-head comparison of these methods has shown that their efficacy depends on the pre-meal scenario and that not one of these methodologies globally outperforms the others [36]. 

Given the complexity of the problem, the nonlinear nature of glucose-insulin dynamics and, the need to utilize information drawn from different domains, suitable data-driven techniques can usefully be adopted to deliver personalized bolus recommendations. Methodologies from the field of machine learning, for example, linear regression techniques, neural networks, and random forests, to name a few, could be used to personalize insulin bolus computation, making use of features extracted from the CGM data stream, such as the ROC information, and other (easily accessible) therapy parameters, such as CR, CF and the insulin pump basal rate [25]. There are two approaches that can be adopted to achieve this scope:

- using the rationale of existing methodologies [33,34,35], correcting the insulin dose provided by the SF, but in a personalized, rather than fixed, manner;
- abandoning the use of the insulin bolus provided by the SF as an initial estimate of the bolus, and instead, designing a new formula for insulin bolus determination that will naturally take into account CGM-derived information and current patient status and characteristics.

In the following two subsections, we present two algorithms developed by our research group at the University of Padova that take these two different approaches. In Section 3.1 we describe a new personalized method, based on a neural network, to adjust the SF by exploiting both CGM information and easily accessible patient parameters. In Section 3.2 we describe a newly developed formula for insulin meal bolus calculation based on a simple, but effective, linear regression model. 

### 3.1. A Neural Network-Based Methodology for Personalized SF Correction

The approach presented in this section attempts to improve the meal insulin bolus calculation by correcting the G_c_ value used in the SF (Equation (1)) of a quantity X that depends on both the pre-meal conditions and the patient’s characteristics. In particular, Cappon et al. [37] used a large training set of simulated data to train a neural network (NN) that predicts the optimal value for X, i.e., the value of X that optimizes post-prandial glucose control. The training set, which spanned a wide range of pre-meal conditions and patient characteristics, was created in silico by the UVa/Padova T1D Simulator [38], a simulation tool that includes a model of glucose-insulin-glucagon dynamics in subjects with T1D, accepted by the FDA to substitute preclinical trials of certain insulin treatments. 

**Figure 2:**
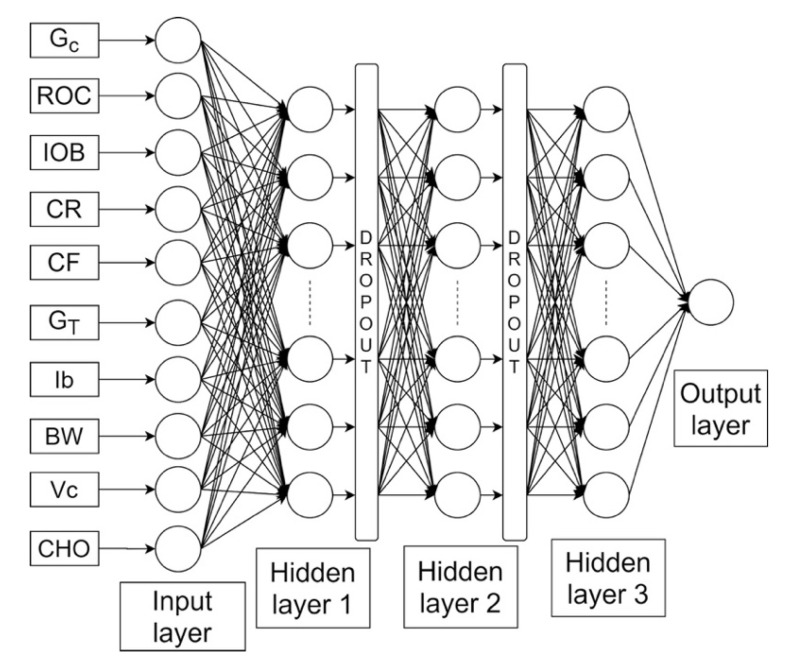
Structure of a NN able to correct meal insulin doses computed by the SF (adapted from Figure 1 of [37]).

The NN developed, shown in Figure 2, is a fully connected feedforward NN, composed of 3 hidden layers whose structure and inputs were chosen after a preliminary analysis, carried out on training data, to obtain the best compromise between model complexity and fitting accuracy. Two dropout layers were inserted between each hidden layer to prevent overfitting and to make the NN capable of generalization. The network input layer consists of 10 neurons fed with 10 features. Three features describe the pre-prandial status of the patient: G_c_, ROC and IOB. The other four features represent the patient’s therapy parameters: CR, CF, G_T_ and the insulin pump basal infusion rate, I_b_. Two features related to the patient’s physiology are also considered: body weight, BW, and the class of the patient’s intra-day insulin sensitivity variability pattern, VC. The last feature represents the meal carbohydrate amount, CHO. The output layer is composed of a single neuron that combines the outputs of the last fully connected layer so as to produce an estimate of the best bolus correction X. 

Training of the NN was performed through the gradient descent RMSprop training algorithm, applied in a mini-batch mode, and stopped after 10 consecutive worsenings in NN performance on the validation set, thus using a cross-validation (CV) procedure. The NN hyper-parameters (i.e., the number of neurons per layer and the activation function type) were tuned using a five-fold CV procedure, in other words, the training set was divided into five groups of data, four of which were used for training the NN and the remaining one used as a validation set to compute the mean squared error (MSE). The optimal set of hyper-parameters was chosen as that giving minimum MSE. We refer the reader to [37] for further details.

The performance of the NN-based BC was assessed on an independent test set generated by the UVa/Padova T1D Simulator in terms of blood glucose risk index (BGRI) [39], i.e., a popular metric of glycemic control that, thanks to symmetrisation of the glucose scale, is equally sensitive to hypoglycemia and hyperglycemia. It condenses the goodness of glycemic control into a single quantity, or risk index, which facilitates interpretation and comparison of the results. The results showed that the NN-based BC achieved significantly lower BGRI values than did the SF. This suggests that the NN-based BC is a promising technique to personalize T1D therapy and provide insulin dose recommendations that are tailored to the subject and the specific pre-meal conditions, by identifying the type of conditions requiring very aggressive, or very limited, interventions. 

Although, on average, the NN-based BC did improve BG control when compared to the SF, in a non-negligible number of cases the dose provided by the NN-based BC led to worse glycemic control than did the SF. While, this highlights a restriction of the domain of validity of the NN, it also suggests that limitations in predicting the optimal dose may be intrinsically related to the strict constraints related to the original structure of the SF. Indeed, the SF is intrinsically suboptimal, and using it as a starting point to be corrected might prove to be an obstacle in the training process of NN [36]. 

### 3.2. New Linear Regression Model for Insulin Dosing

Machine learning techniques could be used to learn new dosing rules from scratch to overcome the limitations that may derive from the constraints imposed by the SF structure. In Noaro et al. [40], a new formula to calculate meal insulin doses was derived using regularized multiple linear regression. This approach, although very simple, is particularly suitable for this purpose because it provides a model in which the relationship between the inputs (i.e., the patient’s characteristics and information on pre-meal conditions) and the output (i.e., the insulin dose) can be interpreted, an aspect that could facilitate acceptance of a new dosing formula by clinicians. 

The model was developed using the data of 100 virtual subjects simulated using the UVa/Padova T1D Simulator. A multitude of different meal conditions in terms of pre-prandial ROC, BG, and meal amounts were considered for each individual, for a total of 162,000 combinations of virtual subjects and meal conditions. For each combination of meal conditions and virtual patients, the optimal meal insulin dose was determined by a grid search and 11 potentially-predictive features were extracted: the variables included in the SF, plus 3 additional features, namely I_b_, BW and ROC. Moreover, a linear basis expansion of the feature set was performed, wherein the vector of inputs was augmented with additional variables, which are obtained by applying polynomial transformations of the features (both quadratic and interaction terms between features were considered).

The dataset was split into independent training and test sets, containing data from different subjects. A multiple linear regression model was trained on the training set, estimating the model coefficients through LASSO regularization. LASSO regularization penalizes large values of the model coefficients and the smaller, in absolute value, coefficients are set to zero. In this way, LASSO regularization implicitly performs feature selection. The final formula included 14 features. The performance of the new formula was assessed on the test set and compared to the SF in terms of BGRI. The results show that the new formula resulted in improved glycemic outcomes, i.e., lower BGRI values, than the SF. Of course, despite the promising results obtained in simulation, clinical trials to prove the safety and the effectiveness of the new formula, are required before it can be adopted in diabetes management.

In conclusion, machine learning methods can be used to design new data-driven formulae for insulin dosing that are able to naturally account for CGM information (including glucose ROC) and which could improve glycemic control in T1D. 

## 4. Adaptive Tuning of Bolus Calculator Parameters Exploiting AI Techniques

One critical point of T1D therapy is tailoring the therapy parameters. CR and CF, for example, are linked to the patient’s insulin sensitivity and need to be periodically adjusted based on a retrospective review of the glucose measurements collected. When a new pattern of glycemic risk is identified by either the clinical team or the patient, new insulin dosing parameters must be calculated and implemented. This is a time-consuming and challenging task, whose automatization would reduce the burden of diabetes management and streamline clinic visits [30].

Researchers are actively working on developing algorithms that automatically adapt insulin therapy parameters to meet individual needs, in particular updating the therapy parameters in the presence of activities that induce changes in insulin sensitivity. In this section, we review some of the state-of-the-art algorithms, based on AI, which have been proposed for this purpose in the literature.

Herrero et al. [41] proposed an approach to automatically adjust CR and CF using CGM measurements. This method updates the parameters values every day in a run-to-run (R2R) fashion. The magnitude of the update is established based on the distance between the minimum postprandial glucose concentration (measured by CGM) and the patient’s target BG concentration. The results obtained from a simulated experiment suggested that R2R offers a promising approach to optimizing SF parameters [41]. 

One fundamental issue of R2R is that it assumes that the process that is to be controlled, in our case the BG level, is repetitive. This assumption is somewhat unrealistic in many cases, thus it limits R2R applicability. More specifically, patients with T1D face events, such as physical exercise, hormone cycles, psychological stress, alcohol consumption and illness that cannot be taken into account using the simple R2R methodology.

**Figure 3:**
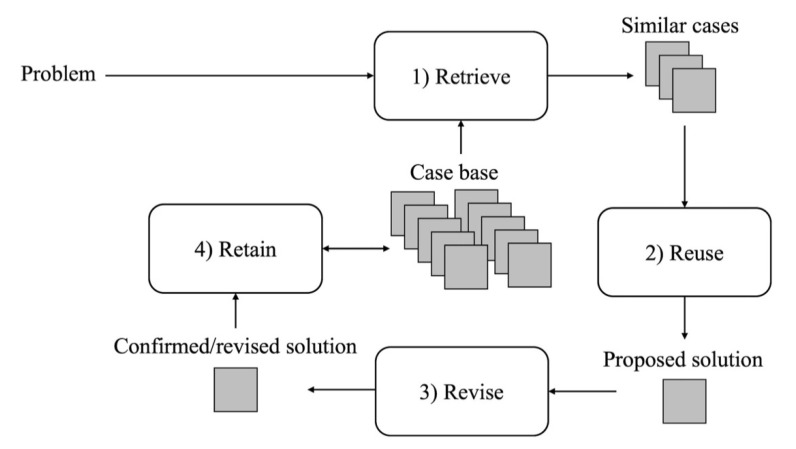
Schematic representation of the CBR cycle proposed by Aamodt and Plaza [43].

One interesting approach which integrates R2R with case-based reasoning (CBR), hereafter referred to as R2R+CBR, was proposed in [42] to overcome this limitation. CBR is an AI technique which solves new problems based on the solutions of similar past problems [43]. As shown in Figure 3, the CBR utilizes a case-base (CB), i.e., a database of previously addressed problems, and includes four steps, namely: Retrieve, Reuse, Revise, Retain [43]. Herrero et al. [42] developed an algorithm in which CBR is used to optimize, in a R2R framework, the CR and CF values to be used in different situations (e.g., meal and patient conditions), thus adding flexibility to the BC. In the general CBR formulation, CB is defined as a set of cases *C_i_*:

(2) $$CB≔\left\{C_{1},\;C_{2},\;\ldots ,\;C_{n}\right\}$$

where a case *C* is a triplet defined as:

(3) C≔{P, S, O}

with *P* representing a set of parameters describing a problem, *S* the solution to the problem and *O* the outcome of applying *S* to solve *P*. In Herrero et al. [42], the problem is how to calculate the insulin dose, which was formalized by considering two parameters: the type of meal T (i.e., breakfast, lunch, or dinner), and the intensity of physical activity E (none, moderate, intense):

(4) P≔{T, E}.

The solution *S* of a case is then defined as the parameters of the bolus calculator that must be optimized:

(5) S≔{CR, CF}.

Lastly, the outcome is defined according to two indicators of post-prandial glucose control,

(6) $$O≔\left\{AUC,\;G_{min}\right\}$$

these indicators are the area under the curve (*AUC*) of the post-prandial glucose profile and the minimum postprandial glucose value (*G_min_*), both of which are calculated using the CGM data collected in the 5-h time-window after the meal.

The CBR’s four steps are implemented in Herrero et al. [42] as follows.

- Step 1. Retrieve: given a target problem retrieve, from the CB, the most similar relevant case for solving it. The similarity between the current case, with parameters *P_c,_* and a case in the CB with parameters *P_k_*, is assessed by a weighted average of the relative differences between the components of *P_c_* and *P_k_*. The case whose parameters are most similar to the current case parameters (i.e., with the lowest relative difference) is retrieved from the CB.
- Step 2. Reuse: map the solution *S* from the retrieved case to the target problem and use it. Once the case has been retrieved, the algorithm extracts the corresponding solution *S* and reuses the associated *CR* and *CF* values to compute the insulin dose to inject using the SF of Equation (1).
- Step 3. Revise: having reused the previous solution to the target situation, assess the performance obtained and, if necessary, revise it. If the glycemic control outcome obtained as a result of the reuse step is not optimal, then the solution *S* is revised. In particular, if the following condition is satisfied,
  (7) $$G_{min}\leq G_{l}$$
  where *G_l_* represents a low BG threshold (e.g., 80 mg/dL), then the *CR* is updated as:
  (8) $$CR_{k+1}=\frac{CR_{k}G_{l}}{G_{min}}.$$
  Otherwise, the *CR* is revised using the following update rule,
  (9) $$CR_{k+1}=CR_{k}+K\cdot \left(AUC_{r}-AUC\right)$$
  where *K* is a tunable gain parameter that represents the aggressiveness of the update, and *AUC_r_* is the target optimal value for *AUC*. After revising the *CR*, the value of *CF* will be updated by multiplying the revised *CR* value for a proportionality constant.
- Step 4. Retain: store the resulting experience in memory. The revised solution is stored in the CB. If the CB already contains a case for the current problem, the corresponding solution is updated with the revised solution, otherwise a new case for the current problem will be created in the CB.

The R2R+CBR algorithm was first tested in simulation and compared with the stand-alone R2R algorithm. A scenario of one-month duration, including patient behaviour and physiological variability was generated using the UVa/Padova T1D Simulator [38]. The results obtained in a cohort of 20 virtual subjects showed that the adoption of R2R+CBR significantly improved glycemic control, achieving better results than the stand-alone R2R algorithm. The proposed R2R+CBR algorithm was able to tackle intrasubject variability and external perturbations, and it was shown to be robust to uncertainty in carbohydrate input (i.e., carb-counting error) and noise in CGM measurements. 

The R2R+CBR algorithm has been implemented in the so-called Advanced Bolus Calculator for Diabetes (ABC4D) [44], which is a novel BC for personalized insulin recommendations for people with T1D. ABC4D was tested in a six-week-long non-randomized clinical study, involving 10 adult subjects with T1D [45]. The study demonstrated the safety and effectiveness of ABC4D, which made it possible to reduce the occurrence of postprandial hypoglycemia.

These encouraging results stimulated further studies on the use of the CBR technique to target BC parameter optimization. Torrent-Fontbona and Beatriz López [46] proposed a new version of the R2R+CBR, whose main novelties are the use of an extended CB and a new mechanism for the reuse step. In this method, a case is defined according to the time of day, estimated carbohydrate intake, pre-prandial glucose concentration, past physical activity, and physical activity planned for the post-prandial phase. Moreover, Torrent-Fontbona and Beatriz López [46] proposed a new strategy for the reuse step. A set of N similar cases is retrieved from the CB and the proposed solution is obtained as the weighted average of the solutions of the N similar cases, with weights proportional to similarity scores. Conversely, in Herrero et al. [42], the proposed solution in the reuse step was copied from the most similar case retrieved from the CB. The results of a three-month in silico experiment showed that the new version of R2R+CBR achieved better glycemic control metrics when it was compared with the initial version by Herrero et al. [42].

Another CBR-based algorithm for BC was proposed by Brown et al. [47]. In this method, BC parameters were updated in a temporal context, thus retrieving and revising the CB by taking into account not only the current encountered case, but also the sequence of past retrieved cases to enhance the retrieve step itself. The results obtained in a six-month in silico study revealed that the proposed algorithm achieved good results in terms of population glycemic outcomes (the BGRI was reduced by up to 27% after three revisions of bolus calculator parameters) [47].

Other AI techniques, not only CBR-based algorithms, have been proposed for BC parameter optimization. Amongst these is the method offered by Sun et al. [48], who proposed an algorithm based on reinforcement learning (i.e., a branch of machine learning that allows a system to develop self-learning capabilities) designed to optimize insulin therapy by simultaneously adapting CR and insulin basal infusion rate. The encouraging results obtained in a simulated study suggest that reinforcement learning could be a promising approach, one able to both personalize insulin therapy parameters and improve glycemic control.

## 5. Glucose Prediction

The availability of dynamic information on glucose concentration from CGM sensors permits not only the detection of adverse events but also their prediction. The most recent CGM sensors adopt very simple prediction algorithms, e.g., linear extrapolation, to predict future interstitial glucose concentrations (e.g., 15–30 min in advance) in real-time and generate predictive alerts when a hypo/hyperglycemic event is foreseen [49]. If provided sufficiently in advance, predictive alerts allow the patient to take suitable countermeasures, e.g., preventive hypo-treatments or correction boluses, to mitigate, or even prevent, such critical episodes [50]. 

Several algorithms for the real-time prediction of future glucose levels have recently been developed, these can be grouped according to different criteria. A first categorization can be made according to the algorithm inputs: some algorithms use CGM data only [51]. While, others use CGM data plus external inputs, such as the amount of ingested carbohydrates, injected insulin and physical activity [52]. These have been shown to enhance the performance of predictions when compared to predictions using only CGM data [53]. 

Population algorithms compute the glucose prediction by using the same model (i.e., structure and/or order) and the same parameter values for all the patients, i.e., without any personalization. This has the practical advantage that model training has to be performed once only, when the algorithm is designed, and the model learning procedure can take advantage of large datasets of CGM traces. The downside of this approach is that it is not customized using the patient’s data. Another option is to develop subject-specific algorithms, where a different model is trained for each subject, in order to take into account the large inter-individual variability characterizing individuals with T1D [52]. 

Lastly, algorithms can also be classified according to the structure of the model used: Some algorithms are based on physiological models, i.e., compartmental models describing a patient’s physiology, while others adopt black-box models, i.e., models describing the relationship between the inputs and the output, which are not interpretable from a physiological point of view [52]. 

As reported in recent systematic reviews [25,54], AI techniques, especially supervised machine learning techniques, are extensively used for building black-box models for glucose prediction. The most used method is NN, but other machine learning methodologies, such as random forest, support vector regression, regression algorithms and deep-learning techniques. For example, long short-term memory (LSTM) networks, are increasingly being adopted. In the two subsections below we review some of the most relevant state-of-the-art approaches for the prediction of interstitial glucose concentration level (Section 5.1) and hypoglycemic events (Section 5.2). For a systematic literature review on these topics, we refer the reader to [25,52,54]. 

### 5.1. Prediction of Interstitial Glucose Concentration Level

**Figure 4:**
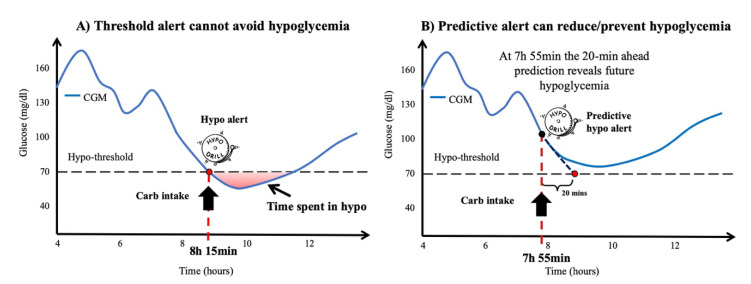
Representative example to illustrate the efficacy of a predictive hypoglycemia alert. (**A**) Imaginary CGM profile of a patient using standard threshold-crossing alerts. A hypo alert is generated at time 8:15 when CGM crosses the hypoglycemia threshold. Even if this patient takes some carbs in response to the alert, the patient spends some time in hypoglycemia. (**B**) Example of how a low predictive alert can help to avoid hypoglycemic events. In this example, the alert is generated at 7:55, when the 20-min ahead glucose prediction crosses the hypoglycemia threshold. If the patient takes carbs in response to the predictive alert, it will be possible to prevent the hypoglycemia.

Most of the prediction algorithms in the literature approach the problem of glucose prediction as a regression problem, in other words they seek to predict the value of interstitial glucose concentration with a prediction horizon (PH) typically below 60 min (30 min is the most frequent PH value) [52]. Interstitial glucose predictions can be used to generate predictive alerts, when for example, the predicted glucose value exceeds a certain high/low glucose threshold, an alarm is generated (Figure 4). 

One of the first AI algorithms for glucose concentration forecasting was proposed by Pérez-Gandía et al. in 2010 [55]. In this work, the authors developed an NN for 30-min ahead glucose prediction based on the CGM data collected in the previous 20-min time-window. In particular, the network architecture had three layers with a first layer of 10 neurons and a second layer of five neurons. These layers had a sigmoidal transfer function, with totally connected and feed-forward neurons. This type of algorithm has recently been included in a prototype of a mobile DSS to support patients with T1D in daily management decisions [56]. More recently, Zecchin et al. [53] have proposed using a jump NN, i.e., an NN with inputs directly connected to both the hidden layer and the output, and with four inputs: CGM reading, its first-order derivative, injected insulin and ingested carbohydrates.

In 2018, a dataset for benchmarking glucose prediction algorithms (the OhioT1DM dataset) was released by Ohio University in combination with The Blood Glucose Level Prediction Challenge (BGLP) at The 3rd Workshop on Knowledge Discovery in Healthcare Data (KDH), whose aim was to compare the efficacy of different machine learning prediction approaches on a standard set of real patient data. In 2020, the dataset was extended in the occasion of the 2nd edition of the BGLP challenge [57]. 

Recently, Xie and Wang compared the performance of several black-box prediction algorithms on the OhioT1DM dataset [58]. In particular, they considered the Autoregressive model with exogenous inputs (ARX), elastic net, LASSO, Ridge and Huber regressions, gradient boosting trees, random forest, support vector regression (SVR) and two deep learning approaches: LSTM and Temporal Convolution Network (TCN). All the methods were assessed considering 30-min PH and different input configurations, i.e., CGM + insulin, CGM + carbs, CGM + insulin + carbs, CGM + insulin + carbs + heart rate, over the past 15, 30, 45, 60 min. The prediction performance was assessed using two commonly used metrics: the root mean squared error (RMSE) between the CGM profile and the predicted profile, and time gain (TG), i.e., the difference between the PH and the delay between the CGM profile and the predicted profile.

The best results were achieved by considering all of the available inputs in a 45-min time-window before the prediction time. The models showing best performance in terms RMSE were ARX, Ridge regression, linear SVR, LSTM, and TCN, with RMSE between 19 and 20 mg/dL. The maximum TG was achieved for Gradient boosting trees and LSTM, which made it possible to predict the CGM data, on average, 7 min in advance. Considering both of the metrics, the models that performed better were linear SVR and TCN with RMSE around 20 mg/dL and a TG of about 6 min.

### 5.2. Hypoglycemic Event Prediction

Most studies on glucose prediction have focused on forecasting future glucose concentration levels using a regression approach, usually with a short PH (e.g., 30 min). However, sometimes in order to prevent the occurrence of certain events more efficiently, a prediction with a larger PH, e.g., 2–3 h, is desirable. For example, consider a scenario in which a patient overestimates the insulin bolus for a meal (for instance, because the patient overestimates meal carbs) and because of the excessive insulin dose experiences hypoglycemia 3 h after the meal. If the patient has used a DSS with a short-term prediction algorithm, a predictive alert might be generated 30–15 min before the event. In this way, the patient would be able to avoid, or mitigate, the event by eating additional carbohydrates. However, the best scenario would be to alert the patient of the risk of post-prandial hypoglycemia at the time of meal bolus, so that the patient could decide to reduce the meal insulin bolus amount, thus avoiding hypoglycemia and the need to consume extra carbs. To implement this strategy, algorithms allowing glucose prediction over a longer PH, e.g., 2–6 h, are required. On the other hand, predicting the exact glucose concentration level over such a long PH would be a very challenging task.

For this reason, some researchers are currently investigating techniques to predict whether or not a specific event (e.g., hypoglycemia) will, or could, occur in a certain time-window, without attempting to predict the exact glucose concentration level. In this case, the glucose prediction problem becomes a classification problem, whereby the outcome could be, for example, a binary variable, with 1 indicating the event occurred and 0 indicating that no event was observed in the predefined time-window. 

Following this approach, Cappon et al. [59] proposed an extreme gradient-boosted tree (XGB)-based algorithm to classify post-prandial glycemic status and a simple empirical rule to adjust meal bolus computation according to the predicted class. The XGB features included glucose readings, meal intakes, insulin administrations, the hour of the day, two binary indicators denoting whether there has been a hypo/hyperglycemic event in the last 3 h, and the time elapsed since the last insulin bolus or meal intake. The results obtained in a virtual cohort of 100 adults, showed that, compared to the SF, the XGB-based BC significantly improved time in euglycemia (from 61.98% to 67.00%) without increasing time in hypoglycemia. 

Reddy et al. [60] proposed two population algorithms for predicting the occurrence of hypoglycemia during aerobic exercise at the beginning of the exercise session. The models considered were a decision tree classifier and a random forest classifier. The features included anthropometric data, physical activity data acquired using a wearable Zephyr heart rate monitor, glucose value at the start of exercise, IOB at the start of exercise, the total daily insulin dosage, and whether glucagon was used within the therapy. The simple decision tree achieved an accuracy of 79.55% with only two features, while the random forest had an accuracy of 86.67%. However, one limitation of this study is that all the data were collected in a controlled inpatient environment. Further validation of the approach with data gathered under free-living conditions, and with different types of exercise, is required.

**Figure 5:**
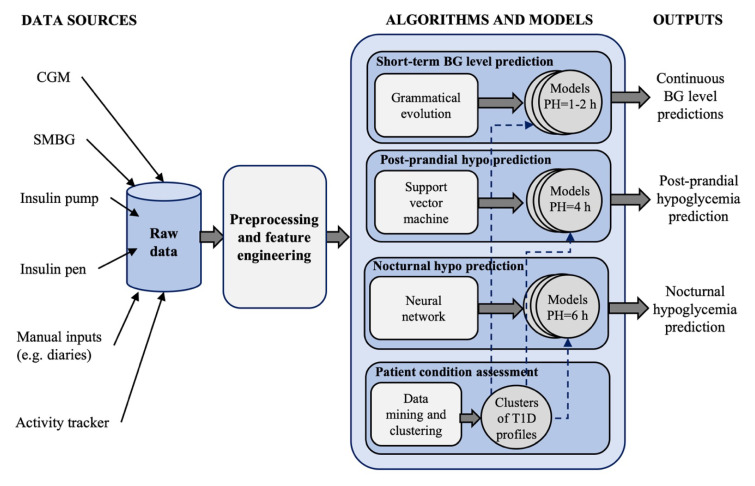
Schematic representation of a proposed Patient Safety System (adapted from Figure 2 of [61]).

Last, Vehì et al. [61] proposed an AI-based DSS, called the Patient Safety System, for the prediction and prevention of hypoglycemic events and the classification of glycemic control profiles. The system is shown in Figure 5 and incorporates 4 modules:

1. A grammatical evolution algorithm for 60-min ahead, personalized, glucose concentration level prediction, that uses, as input features, the CGM data, the IOB and the carbohydrates rate-of-appearance over the past 2 h [62].
2. A personalized support vector classifier for predicting, at meal time, the occurrence of hypoglycemia in the time-window of 4 h after the meal [63]. Nine features are used, including five features extracted from the last hour of CGM data, the basal insulin dose taken in the last 2 h and programed for the next 4 h, the insulin dose to be administered for current meal and the carbohydrates of current meal.
3. An individualized model based on NN for predicting, while the patient is going to bed, if nocturnal hypoglycemia will occur in the next 6 h. The features of this module are: CGM value at sleep announcement, BG control metrics in the last 6 h, CGM ROC during the previous 30 min, the values of the IOB, activity on-board, and remaining carbohydrates rate-of-appearance at sleep announcement.
4. A patient assessment module that uses a data mining algorithm for unsupervised clustering of glucose profiles. The aim of this module is to identify the most common situations affecting BG control for a single patient, information that could be used to further customize the models used by the modules 1–3 (e.g., training the models for each cluster of patient’s data).

Although each module has been independently validated on retrospective data, a clinical validation of the proposed DSS is needed to prove its efficacy as a tool for improving glycemic control in subjects with T1D.

## 6. Conclusions

In managing T1D, the increased availability of internet-connected and interoperable sensors makes it possible to construct large databases, consisting of interstitial glucose concentration measurements, integrated with several other data sources. For example, CGM data can be merged with information provided by other medical devices (SMBG, pumps, smart pens) [64], mobile apps, clinical registries, electronic health records, as well as other wearable sensors, such as activity trackers. The data collected by activity trackers can be used to automatically detect exercise sessions [65], predict exercise-induced hypoglycemia [60], and if necessary, recommend the consumption of carbohydrates to avoid hypoglycemia. Such data integration could contribute to generating a digital ecosystem of diabetes data that can be used to extract new medical knowledge that cannot be discovered by relying on a single source of information [66,67]. Large diabetes databases, integrating multiple data sources, increasingly require the use of AI techniques for the development of applications for therapy personalization and the prevention of complications. AI techniques, indeed, can handle big datasets with different types of data, without the need of an underlying physiological model. Amongst the possible applications, AI-enabled DSSs are promising tools that could potentially reduce the patient burden in diabetes management and improve glucose control. 

In this review, we provided an overview of current research and development trends about the use of AI techniques in DSSs for patients with T1D. We discussed in detail some of the most relevant AI-based approaches that were proposed for three fundamental tasks of DSSs, i.e., personalized insulin bolus calculation, adaptive tuning of bolus calculator parameters and glucose prediction. Currently available literature evidence suggests that AI techniques can achieve very promising performance in these three tasks. However, most of these techniques were validated only in silico or on retrospective real data. A prospective clinical validation proving the safety and effectiveness of such techniques is required to support the adoption of AI-based DSS applications in T1D management.
